# Genetic Characterization of the Apple Germplasm Collection in Central Italy: The Value of Local Varieties

**DOI:** 10.3389/fpls.2018.01460

**Published:** 2018-10-10

**Authors:** Gianpiero Marconi, Nicoletta Ferradini, Luigi Russi, Luciano Concezzi, Fabio Veronesi, Emidio Albertini

**Affiliations:** ^1^Dipartimento di Scienze Agrarie, Alimentari ed Ambientali, Università degli Studi di Perugia, Perugia, Italy; ^2^3a Parco Tecnologico Agroalimentare dell’Umbria, Perugia, Italy

**Keywords:** *Malus × domestica*, SSR markers, local varieties, genetic resources, germplasm collection

## Abstract

In the last 50 years, intensive farming systems have been boosted by modern agricultural techniques and newly bred cultivars. The massive use of few and related cultivars has dramatically reduced the apple genetic diversity of local varieties, confined to marginal areas. In Central Italy a limited spread of intensive fruit orchards has made it possible to preserve much of the local genetic diversity, but at the same time the coexistence of both modern and ancient varieties has generated some confusion. The characterization and clarification of possible synonyms, homonyms, and/or labeling errors in old local genetic resources is an issue in the conservation and management of living collections. 175 accessions provided by 10 apple collections, mainly local varieties, some of unknown origin, and well-known modern and ancient varieties, were studied by using 19 SSRs, analyzed by STRUCTURE, Ward’s clustering and parentage analysis. We were able to identify 25 duplicates, 9 synonyms, and 9 homonyms. As many as 37 unknown accession were assigned to well known local or commercial varieties. Polyploids made up 20%. Some markers were found to be significantly correlated with morphological traits and the *loci* associated with the fruit over color were related to QTLs for resistance to biotic stresses, aroma compounds, stiffness, and acidity. In conclusion the gene pool of Central Italy seems to be rather consistent and highly differentiated compared with other European studies (*F*_ST_ = 0.147). The importance of safeguarding this diversity and the impact on the management of the germplasm living collection is discussed.

## Introduction

Apple (*Malus × domestica* Borkh., family Rosaceae, tribe Pyreae, 2*n* = 2*x* = 34) is one of the most ancient and widespread fruit crops in temperate regions. Almost certainly the domesticated apple is the result of a long evolutionary process extending over thousands of years, and it seems several species have contributed to its gene pool. On the basis of genetic data, of fruit and tree morphology, the wild Asian species *M. sieversii* M. Roem is actually considered the main contributor to the *Malus × domestica* gene pool, and the Tian Shan Mountains (Central Asia) the center of origin (Velasco et al., 2010; Cornille et al., 2014). Furthermore, hybridizations with other wild apple species present along the Silk Route, such as *M. baccata* (L.) Borkh. in Siberia, *M. orientalis* Uglitz. in Caucasus and *M. silvestris* (L.) Miller in Europe, have produced the diversity currently present in the domesticated apple (Vavilov, 1926; Harris et al., 2002; Cornille et al., 2012, 2014; Gross et al., 2014). Despite high genetic variability, the thousands of cultivars distributed throughout the world and the world-wide breeding programs, mainly based on organoleptic traits, aesthetic standards and disease resistance, the size of the genetic resources used by breeders has been limited and reduced to a few varieties such as ‘Cox’s Orange Pippin,’ ‘Golden Delicious,’ ‘Jonathan,’ ‘Red Delicious,’ and ‘McIntosh’ (Noiton and Alspach, 1996). As a result, apple cultivation today is limited to closely related cultivars, and four of them, namely Golden Delicious, Gala, Red Delicious, and Idared, account for 48% of global production. Actually, the most important variety is Golden Delicious with 2.546 million metric tons, followed, by Gala with 1.331 million metric tons and Idared with 1.111 million metric tons (Food and Agricultural Organization of the United Nation [FAO], 2016; WAPA, 2017). This massive use of limited and related cultivars, combined with vegetative practices based on cuttings and grafting, has dramatically reduced apple genetic diversity and, hence, many interesting and well adapted traditional and local varieties, considered obsolete, were no longer cultivated and have been partly lost (Hammer et al., 2003).

Similar trends occurred in Italy, which with its 2.5 million t represents the fifth apple producer in the World and the second in Europe (Food and Agricultural Organization of the United Nation [FAO], 2016). Apple production is mainly concentrated in the North of Italy, in particular in Trentino Alto Adige, which with its 1.7 million t represents 67% of total Italian production, followed by Veneto (11%), Piemonte (6%), and Emilia-Romagna (6%). In these regions, as well as in the World and in other European Countries, production is based mainly on intensive orchards with few commercial varieties: Golden Delicious, Gala, Red Delicious, Fuji, and Granny Smith (Assomela-CSO, 2017). In Center-South Italy intensive apple orchards are appreciably present only in Campania (3% of Italian production). Historically these areas have never been inclined to intensive cultivation of fruit in general and apples in particular, even if in the 1920s there were unsuccessful attempts to introduce improved varieties in intensive orchards (Albertini et al., 2015). In these regions, apart from few family-run orchards, fruit production was, and currently remains, mainly directed toward self-consumption and local markets. Therefore, in Central Italy the almost complete absence of intensive apple cultivation allowed for the preservation of much of the existing genetic diversity even if the limited coexistence between modern and local varieties and the evolution of the farming systems since the 1950s, has led to the disappearance of several interesting and well adapted ancient varieties. Many of these varieties, although of low productivity, were relatively stable under extreme environmental conditions, and their high genetic variability guaranteed reliable harvesting for local communities in the past (Albertini et al., 2015). Over time, some of the modern, introduced varieties mixed with the autochthones, increasing the panorama of choice on the one hand, but generating some confusion regarding local genetic resources and their correct denomination on the other. Consequently, the need for characterization and clarification of possible synonyms, homonyms, and/or labeling errors in these old and local genetic resources is a fundamental and necessary step for the conservation and management of living collections. Indeed, the genetic variability and allelic diversity present in these old accessions could be of extreme interest in terms of response to selection in adaptation toward a changing environment (Caballero and García-Dorado, 2013). Therefore, even though such varieties are characterized by low fruit quality and yield, their allelic diversity could be essential for crop improvement, providing the presence of interesting traits for the development of new varieties.

In this scenario, molecular markers can provide a valid tool to assess genetic diversity, uncovering duplicates or possible synonymies and/or homonymies, and helping the management of plant genetic resources. Several studies based on molecular markers have assessed the diversity in *Malus*, including modern cultivars and old, local germplasm accessions (Pereira-Lorenzo et al., 2007, 2008, 2017; Gharghani et al., 2009; Gasi et al., 2010; van Treuren et al., 2010; Gross et al., 2012; Urrestarazu et al., 2012; Gao et al., 2015; Liang et al., 2015; Lassois et al., 2016; Vanderzande et al., 2017). For these purposes microsatellites (SSRs) are considered the marker of choice due their high polymorphism and co-dominant inheritance, widely used to asses the genetic diversity at population and individual level in many plant core collections (Blair et al., 2009; Zhang et al., 2011; Patzak et al., 2012; Emanuelli et al., 2013; Urrestarazu et al., 2016; Mousavi et al., 2017). To date several hundred SSR markers have been developed and mapped in apples by Guilford et al. (1997), Gianfranceschi et al. (1998), Liebhard et al. (2002), Vinatzer et al. (2004), Silfverberg-Dilworth et al. (2006), Han and Korban (2008), and Celton et al. (2009). Many of these mapped SSR are linked to QTL with interesting agronomical, morphological, and organoleptic traits and could be used as molecular tools for marker assisted selection (MAS) in plant breeding programs (Gianfranceschi et al., 1998; Gygax et al., 2004; Kunihisa et al., 2014; Sun et al., 2014; Liu et al., 2016). Microsatellites have been also widely used in apples to asses the genetic diversity in core collections (Yun et al., 2015; Lassois et al., 2016; Urrestarazu et al., 2016), cultivar characterization (Goulão and Oliveira, 2001; Patzak et al., 2012; Pérez-Romero et al., 2015), and parentage analysis (Kitahara et al., 2005; Moriya et al., 2011; Lassois et al., 2016).

The present research aims to understand the relationship between local accessions in central Italy with old and new varieties, as well as investigate for synonyms/homonyms for a better management of conservation and propagation of genetic resources.

## Materials and Methods

### Plant Material

One hundred and seventy five accessions of *Malus × domestica*, mostly Italian, were included in this study and were provided by several collections: the National Center of Fruit Tree Germplasm (CREA, coded c01), Parco Tecnologico Agroalimentare dell’Umbria (3A-PTA, coded c02 and c03 as coming from two living collections), the Department of Agriculture, Forestry and Food Science of the University of Torino (DISAFA, c04), the Archeologia Arborea private collection (c05), the Department of Agricultural, Food and Environmental Sciences of the Polytechnic University of Marche (D3A, c06), Malva Rinaldi School in Torino (c07), the Department of Agricultural Science, University of Bologna (DipSA, c08), the Giardino Armonico of Bevagna private collection (c09) and Azienda Ortofrutticola Sett’Olmi, Perugia (c10). Details are reported in **Table 1**. Many of the 175 accessions were well documented by reliable historical sources. Those lacking of several reliable information were coded as Unknown. Therefore, based on the initial information, the 175 accessions used in this study were classified into 17 commercial varieties (CV) used as control, 99 local varieties (LV), and 59 unknown accessions (UA).

**Table 1:** Collection code^1^, accession name, ploidy^2^ (D, diploids; P, polyploids) and status (LV, local variety; CV, commercial variety; UA, unknown) of 175 apple accessions used in the present study.

Coll. code	Accession name	Ploidy	Status
c01	001_Cerina	D	LV
c01	002_Zuccherina	D	LV
c01	003_Gelato Cola	D	LV
c01	004_Ghiacciola	P	LV
c01	005_Pom de L’oio Rosso	D	LV
c01	006_’E Santu Giuanni	D	LV
c01	007_’E Santu Giuanni Rossa	D	LV
c01	008_Roggia	D	LV
c01	009_Ruzine	D	LV
c01	010_Ruzza	D	LV
c01	011_Sona	D	LV
c02	012_Ruzza	D	LV
c02	013_San Giovanni	D	LV
c02	014_Oleosa	D	LV
c02	015_A Sonagli	D	LV
c02	016_Gelata	D	LV
c02	017_Cera	D	LV
c03	018_Roggia	D	LV
c04	019_Gris d’la composta	D	LV
c04	020_Gris canavoeit	D	LV
c04	021_San Sebastian	P	LV
c04	022_Ruggine piatta	D	LV
c04	023_Buras	P	LV
c04	024_Grigia di Torriana	D	LV
c05	025_Oliata	P	LV
c05	026_Diacciata	P	LV
c06	027_Oleata	D	LV
c06	028_Cerina	D	LV
c06	029_Gelata	D	LV
c06	030_Gelata	D	LV
c02	031_Olia	D	LV
c02	032_Casciola	P	LV
c02	033_Panaia	D	LV
c02	034_Panaia	P	LV
c02	035_Pagliaccia	P	LV
c02	036_Casciola	P	LV
c02	037_Casciola	D	LV
c07	038_Sonaja Rossa	D	LV
c07	039_Ciocarina Bianca	D	LV
c07	040_Ciocarina Rossa Dossa	D	LV
c07	041_Ciochera Rosa	D	LV
c08	042_Pum Giuan	D	CV
c08	043_San Giovanni PT	D	LV
c08	044_San Giovanni MO	P	LV
c08	045_San Giovanni BO	P	LV
c08	046_Ceres	D	LV
c02	047_Golden Delicious	D	CV
c02	048_Golden Gala	D	CV
c02	049_Amerina	D	LV
c02	050_Pianella	P	LV
c02	051_Unknown	D	UA
c02	052_Appiola Rossa	D	LV
c02	053_Rosa D’Amelia	D	LV
c02	054_Unknown	P	UA
c02	055_Unknown	D	UA
c02	056_Unknown	P	UA
c02	057_Bianchina	D	LV
c02	058_Coccianese	D	LV
c02	059_Coccianese	D	LV
c02	060_Unknown	D	UA
c02	061_Limoncella	D	LV
c02	062_Piattuccia	D	LV
c02	063_Stratarina	D	LV
c02	064_Conventina	D	LV
c02	065_Muso di Bue	D	LV
c02	066_Ruzza	D	LV
c02	067_Spoletina	P	LV
c02	068_Unknown	D	UA
c02	069_Unknown	P	UA
c02	070_Rossa Doglio	D	LV
c02	071_Gialla Doglio	D	LV
c02	072_Ciucca Dolcetta	D	LV
c02	073_Polsola	P	LV
c02	074_Dolcetta	D	LV
c02	075_Unknown	D	UA
c02	076_Unknown	D	UA
c02	077_Appiola Rossa	P	LV
c02	078_Appiola	D	LV
c02	079_Unknown	D	UA
c02	080_Ducale	D	LV
c02	081_Pianella	D	LV
c02	082_Rosciola	D	LV
c02	083_Unknown	P	UA
c02	084_Unknown	D	UA
c02	085_Unknown	D	UA
c02	086_Ulpia	D	LV
c02	087_Amerina	D	LV
c02	088_Saragano Rossa	D	LV
c02	089_Unknown	D	UA
c02	090_Unknown	D	UA
c02	091_Saragano Gialla	D	LV
c02	092_Unknown	D	UA
c02	093_Unknown	P	UA
c02	094_Unknown	D	UA
c02	095_Unknown	D	UA
c02	096_Unknown	D	UA
c02	097_Unknown	D	UA
c02	098_Unknown	D	UA
c02	099_Unknown	P	UA
c02	100_Unknown	D	UA
c02	101_Maggiolina	D	LV
c02	102_Unknown	P	UA
c02	103_Unknown	D	UA
c02	104_Rossa Montelupone	D	LV
c02	105_Unknown	D	UA
c02	106_Unknown	D	UA
c02	107_Gialla Montelupone	D	LV
c02	108_Unknown	D	UA
c02	109_Unknown	P	UA
c02	110_Unknown	D	UA
c02	111_Unknown	D	UA
c02	112_Paradisa	D	LV
c02	113_Unknown	D	UA
c02	114_Unknown	P	UA
c02	115_Coppiola	D	LV
c02	116_Unknown	P	UA
c02	117_Unknown	D	UA
c03	118_Rosa in Pietra	D	LV
c03	119_Del Castagno	D	LV
c03	120_Ciucca	D	LV
c03	121_Rosa Gentile	D	LV
c03	122_Rosa Romana	P	LV
c03	123_Polsola	D	LV
c03	124_Bianchina	P	LV
c03	125_Unknown	D	UA
c03	126_Limoncella	D	LV
c03	127_Conventina	D	LV
c03	128_Roggia	D	LV
c09	129_Durello	D	LV
c09	130_Calvilla d’Estate	D	CV
c09	131_Reinette du Canada	P	CV
c09	132_Reinette Ananas	D	CV
c09	133_Reinette de Champagne	D	CV
c09	134_Limoncina	D	CV
c09	135_Decio	D	LV
c09	136_Annurca	D	CV
c09	137_Abbondanza	D	CV
c02	138_Unknown	D	UA
c02	139_Spoletina	P	LV
c02	140_Unknown	P	UA
c02	141_Limoncella	D	LV
c02	142_Unknown	D	UA
c02	143_Unknown	D	UA
c02	144_Unknown	D	UA
c02	145_Unknown	P	UA
c02	146_Gelata	D	LV
c02	147_Rosa in Pietra	D	LV
c02	148_Unknown	D	UA
c02	149_Coccianese	D	LV
c02	150_Unknown	D	UA
c02	151_Unknown	D	UA
c02	152_Cera	D	LV
c02	153_Unknown	D	UA
c02	154_Unknown	D	UA
c02	155_Unknown	D	UA
c02	156_Statia	D	LV
c02	157_Unknown	D	UA
c02	158_Paonazza di Piubbica	D	LV
c02	159_Annurca	D	CV
c02	160_Unknown	D	UA
c02	161_Unknown	P	UA
c02	162_Unknown	P	UA
c02	163_Unknown	P	UA
c02	164_Unknown	D	UA
c02	165_Rossa di San Venanzo	D	CV
c02	166_Unknown	D	UA
c02	167_Unknown	D	UA
c02	168_Unknown	D	UA
c02	169_Rosona	P	LV
c10	170_Golden Clone B	D	CV
c10	171_Fuji	D	CV
c10	172_Stark Delicious	D	CV
c10	173_Unknown	D	UA
c10	174_Gold Chief (Gold Pink)	D	CV
c10	175_Cripps Pink	D	CV

*^*1*^c01 = CREA, National Centre of Fruit Tree Germplasm; c02 = 3A-PTA, Parco Tecnologico Agroalimentare dell’Umbria, Pantalla; c03 = 3A-PTA, Casalina; c04 = DISAFA, Department of Agriculture, Forestry and Food Science, University of Torino; c05 = Archeologia Arborea, private collection; c06 = D3A, Department of Agricultural, Food and Environmental Sciences, Marche Polytechnic University; c07 = School Malva Rinaldi of Torino; c08 = DipSA, Department of Agricultural Science, University of Bologna; c09 = Giardino Armonico of Bevagna, private collection; c10 = Nursery Sett’Olmi, private collection. ^*2*^An accession is considered polyploid when at least 3 out of 19 loci showed a third allele.*

### Microsatellite Amplification

Total genomic DNA was purified from young leaves using the DNeasy 96 Plant Kit (Qiagen) according to manufacturer’s protocol. Twenty one apple SSR primer pairs (Liebhard et al., 2002; Vinatzer et al., 2004; Silfverberg-Dilworth et al., 2005) distributed over the 17 apple linkage groups were used (**Table 2**). Primer sequence and allele range for validated *loci* were analyzed by Multiplex Manager (Holleley and Geerts, 2009) to determine the best sets of *loci* to combine in a multiplex protocol. Multiplex Manager was used with the option of grouping all validated *loci* within the minimum number of PCRs avoiding allele range overlap and primer interactions.

**Table 2:** Characteristics of the 19 SSR markers used in the study, forward and reverse sequences, repetitive motives and types (Perfect, Imperfect, Compound), and linkage group.

Locus	Forward seq. (5′– 3′)	Reverse seq. (5′- 3′)	Rep. motiv	Rep. type	Linkage group
CH05c06	atcaacagtagtggtagccgggt	attggaactctccgtattgtgc	CT	Imp	16
CH-Vf1	atcaacacgagcagcaaag	catacaaatcaaagcacaaccc	AG	Imp	1
Hi07h02	caaattggcaactgggtctg	gtttaggtggaggtgaagggatg	GT	Perf	17
CH03d12	gcccagaagcaataagtaaacc	attgctccatgcataaaggg	CT	Comp	6
CH05e03	cgaatattttcactctgactggg	caagttgttgtactgctccgac	GA	Perf	2
Hi04e04	gaccacgaagcgctgttaag	gtttcggtaattccttccatcttg	GA	Perf	16
CH02b03b	ataaggatacaaaaaccctacacag	gacatgtttggttgaaaacttg	CT	Perf	10
CH01f03b	gagaagcaaatgcaaaaccc	ctccccggctcctattctac	GA	Imp	9
CH02c09	ttatgtaccaactttgctaacctc	agaagcagcagaggaggatg	CT	Perf	15
Hi22f12	ggccctcacccagtctacatt	gtttggtgtgatggggtactttgc	CTT	Imp	5
CH01g12	cccaccaatcaaaaatcacc	tgaagtatggtggtgcgttc	CT	Imp	12
AU223657	ttctccgtccccttcaacta	caccttgaggcctctgtagc	CT	Imp	3
CH01h02	agagcttcgagcttcgtttg	atcttttggtgctcccacac	CT	Imp	9
CH01h01	gaaagacttgcagtgggagc	ggagtgggtttgagaaggtt	CT	Perf	17
CH04a12	cagcctgcaactgcacttat	atccatggtcccataaacca	CT	Imp	11
Hi03a10	ggacctgcttccccttattc	cagggaacttgttgatgg	GA	Imp	7
CH04c07	ggccttccatgtctcagaag	cctcatgccctccactaaca	GA	Perf	14
CH01c06	ttccccatcatcgatctctc	aaactgaagccatgagggc	GA	Perf	8
CH02g01	gatgacgtcggcaggtaaag	caaccaacagctctgcaatc	CT	Perf	13
CH03d07	caaatcaatgcaaaactgtca	ggcttctggccatgatttta	CT	Perf	6
Hi23g02	ttttccaggatatactacccttcc	gtttcttcgaggtcagggtttg	CA	Perf	4

PCRs were carried out in a final volume of 25 μl using 1 × Type-it Microsatellite PCR Master Mix (Qiagen), 0.2 μM of each fluorescent forward primer labeled with 6-FAM or ROX dyes (Sigma) and reverse unlabeled primer and 20 ng of template DNA. All amplifications were performed in a GeneAmpPCRSystem 9700 (Applied Biosystems, United States) consisting of a denaturing step of 5 min at 95°C followed by 30 cycles of 95°C for 30 s, 57°C for 90 s and 72°C for 30 s, and a final elongation step of 30 min at 60°C.

PCR products were separated and analyzed on a 3130 XL DNA Analyzer (Applied Biosystems). The size of the amplified products was determined on internal standard DNA (GeneScan 500 Liz, Thermo) and the scorable peaks were assigned by GeneMapper software v.4.0 (Applied Biosystems).

### Morphological Characterization

Phenological and morphological traits of interest were scored as follows: time of eating maturity (1 = early summer, 3 = late summer-early autumn, 5 = autumn, 7 = winter); fruit shape (1 = cylindrical waisted, 2 = conic, 3 = ovoid, 4 = cylindrical, 5 = ellipsoid, 6 = globose, 7 = obloid); fruit ground color (1 = green, 3 = yellow, 5 = red); hue over color of fruit (1 = absent, 3 = yellow, 5 = orange, 7 = pink, 9 = red); fruit rustiness (1 = absent, 9 present), pulp color (1 = white, 3 = cream, 5 = yellow, 7 = red). The scores were assigned by 3 operators who exchanged opinions before eventually providing individual scores, which were averaged prior to statistical analysis. Data were available only for 130 accessions and were therefore used primarily to ascertain doubts (**Supplementary Table S1**).

### Data Analysis

For each *locus*, common PCR artifacts leading to genotyping error were investigated. Presence of null alleles, large allele dropout and extreme stuttering was inferred by means of bootstrapping in Micro-Checker v2.2.3 (Van Oosterhout et al., 2004) based on 1000 bootstraps and 95% confidence interval. A preliminary analysis detected a deviation due the presence of null alleles for two *loci* (Hi23g02 and Hi03g02) which were therefore discarded. The statistical analysis was then based on the remaining 19 *loci* (**Supplementary Table S2**).

The statistical analysis of the SSR data matrix was carried out by Genodive (Meirmans and Van Tienderen, 2004) and SPAGeDi1.2 (Hardy and Vekemans, 2002). Both software packages are able to analyze data files containing diploid and polyploid accessions together. The analysis included: the detection of the number of allele (Na) per *locus*, the effective number of alleles (Ne), the percentage of rare alleles (RA = allele frequency < 0.01), of the observed (H_o_) and expected (H_e_) heterozygosity (Nei, 1978), inbreeding coefficient F, polymorphic information content (PIC) (Botstein et al., 1980) at each *locus*, determined using the following equation:

$$\begin{array}{c} PIC=1-\sum_{i=1}^{n}p_{i}^{2}-{\left(\sum_{i=1}^{n}p_{i}^{2}\right)}^{2}+\sum_{i=1}^{n}p_{i}^{4} \end{array}$$

The analysis also included the probability of identity (P_ID_) (Waits et al., 2001) and the probability of identity among sibs P_(ID)sib_ (Evett and Weir, 1998), calculated as follows:

$$\begin{array}{c} P_{\mathrm{ID}}=\sum p_{i}^{4}+\sum \sum {\left({2p}_{i}p_{j}\right)}^{2} \end{array}$$

$$\begin{array}{c} P_{(\mathrm{ID})\mathrm{sib}}=0.25+\left(0.5\sum p_{i}^{2}\right)+\left[0.5{\left(\sum p_{i}^{2}\right)}^{2}\right]-\left(0.25\sum p_{i}^{4}\right) \end{array}$$

where p_i_ and p_j_ are the frequencies of the ith and jth alleles and i ≠ j.

Finally, the ability of each marker to discriminate two random cultivars was estimated by the Power of Discrimination (PD = 1 - P_ID_) (Kloosterman et al., 1993).

In order to run a cluster analysis on diploid and polyploid accessions together, SSR data were converted to a binary data matrix by assigning “1” to the presence of a defined allele and “0” to its absence. The binary data matrix was then used to estimate a distance matrix, and the 175 accessions were clustered by Ward’s hierarchical method (Ward, 1963) and validated by 1000 bootstrap replicates using PAST software (Hammer et al., 2001). The analysis revealed the presence of several identical accessions with the same genetic profile. As a consequence, these were removed and the remaining 150 were used again as binary data for a cluster analysis based on Ward’s method, and the results compared with the Bayesian model-based clustering of STRUCTURE ver. 2.2.3 (Pritchard et al., 2000) using codominant data based on allele size. STRUCTURE software implements a clustering method assigning individuals and predefined populations to *K* inferred clusters, each characterized by a set of allele frequencies at Hardy-Weinberg equilibrium, based on estimates of the corresponding probabilities of membership to each group. The analyses were run on an admixture ancestral model with correlated allele frequencies, and the number of *K* clusters was determined firstly by simulating a range of *K*-values from 1 to 21 with 10 independent runs each. Since after *K =* 5 there were no other appreciable peaks, STRUCTURE was run again with twenty runs for *K*-values ranging from 1 to 11, using a burn-in and a run length of the Monte Carlo Markov Chain (MCMC) of 300,000 and 500,000 iterations for data collection. The best *K*-value was determined through the Δ*K* method (Evanno et al., 2005) by using the STRUCTURE HARVESTER ver. 0.6.193 website (Earl and vonHoldt, 2012). The genotypes were assigned to the groups according to their highest membership coefficient, considering a strong affinity when the assigning probability (*qI*) was ≥ 0.80 (Breton et al., 2008; Pereira-Lorenzo et al., 2008; Miranda et al., 2010; Urrestarazu et al., 2012). The software STRUCTURE is able to infer the genetic structure either in diploid or polyploid genotypes as described in using the recessive allele approach (Falush et al., 2007). The membership of each accession to the Ward’s clusters and to the groups of STRUCTURE were compared and discussed.

The 92 accessions whose probability (*qI*) was ≥ 0.80 were grouped into *K* = 5 subset of 7, 9, 29, 11, and 36 individuals. The goodness of fit of these 5 groups was investigated by the F statistics (F_IS_ and F_ST_) (Weir and Cockerham, 1984), and the analysis of molecular variance (AMOVA) that estimates the fraction of the genetic variation among and within populations (Excoffier et al., 1992; Michalakis and Excoffier, 1996).

The software FaMoz (Gerber et al., 2003) was used to carry out a parentage analysis, to look for possible genetic relationships (parents) present among the entire sample of accessions and eventually confirming the results of STRUCTURE and Ward’s clustering. FaMoz calculates the logarithm of the likelihood ratio, log of odds ratio (LOD score), by determining the likelihood of an individual being the parent of a given offspring, divided by the likelihood of these individuals being unrelated (Meagher and Thompson, 1986). LOD scores for any potential parentage relationship with a value greater than zero were computed, giving statistical significance to the data. Possible parents determined by LOD scores and significance thresholds were probed among the 150 accessions characterized with the set of 19 SSRs. Through 100,000 simulations with a rate of mistyping errors of 0.1% as described by Gerber et al. (2000), a LOD score threshold of 5.0 was found and used in our work (**Supplementary Figure S1**). For polyploid accessions a FaMoz control analysis was run on a 0–1 data matrix.

Moreover, in order to look for correlations between molecular data and morphological traits, a non-parametric correlation analysis (Spearman) was carried out using SAS 9.1 (Cary, NC, United States).

## Results

### Genetic Diversity

The 19 nuclear SSRs were all polymorphic and produced scorable amplicons with a total of 278 alleles. The average number of alleles per *locus* was 14.6, ranging from 5 (Hi22f12) to 26 (CH05e03), but the number of effective alleles per *locus* was significantly lower (Ne = 5.94) (**Table 3**). With the exception of *locus* Hi22f12, all *loci* showed at least one genotype with three alleles. *Loci* CH02b03b and CH01h01 identified as many as 28 individuals with three alleles, while the other *loci* identified between 8 and 22 individuals. Even if several individuals showed one *locus* with a third allele, only those showing a third allele in at least 3 *loci* (Urrestarazu et al., 2012) were considered putative polyploids. In the present study 35 individuals (20%) were classified as polyploids (**Table 1**), as they showed a third allele from 4 up to 13 *loci*.

**Table 3:** Genetic diversity in terms of range of allele size (bp), number of allele (Na), effective number of alleles (Ne), percentage of rarity (RA), observed (Ho) and expected (He) heterozygosity, inbreeding coefficient (F), polymorphic information content (PIC) and probability of identity (P_ID_ and P_IDsib_) of all 175 accessions of apple germplasm evaluated.

Locus	Range of allele size (bp)	Na	Ne	RA^†^	Ho	He	*F*	PIC	P_ID_^‡^ unrelated	P_IDsib_^‡^
CH05c06	104–134	14	4.68	28.6	0.80	0.79	–0.018	0.784	0.0183	0.3626
CH-Vf1	127–173	14	2.85	57.1	0.75	0.65	–0.154	0.647	0.0761	0.4453
Hi07h02	238–276	17	8.84	23.5	0.86	0.89	0.034	0.885	0.0025	0.3084
CH03d12	96–157	25	7.36	40.0	0.83	0.86	0.041	0.862	0.0091	0.3214
CH05e03	145–224	26	7.79	42.3	0.70	0.87	0.200	0.869	0.0059	0.3169
Hi04e04	208–249	15	6.75	20.0	0.87	0.85	–0.020	0.849	0.0088	0.3275
CH02b03b	73–106	13	6.43	15.4	0.90	0.84	–0.062	0.842	0.0074	0.3308
CH01f03b	137–185	11	4.83	18.2	0.88	0.79	–0.110	0.791	0.0163	0.3587
CH02c09	234–259	12	5.51	16.7	0.82	0.82	–0.005	0.816	0.0139	0.3453
Hi22f12	202–218	5	3.91	–	0.16	0.74	0.784	0.742	0.0307	0.3867
CH01g12	102–186	21	7.77	33.3	0.91	0.87	–0.043	0.869	0.0052	0.3169
AU223657	224–237	6	4.38	–	0.72	0.77	0.067	0.769	0.0180	0.3698
CH01h02	236–255	9	3.36	22.2	0.77	0.70	–0.098	0.701	0.0595	0.4146
CH01h01	104–131	12	6.70	25.0	0.85	0.85	0.000	0.849	0.0050	0.3270
CH04a12	159–202	18	4.53	27.8	0.81	0.78	–0.041	0.777	0.0322	0.3696
Hi03a10	199–291	16	8.89	18.8	0.75	0.89	0.156	0.885	0.0023	0.3080
CH04c07	95–139	16	8.06	12.5	0.91	0.88	–0.038	0.874	0.0039	0.3142
CH01c06	149–191	14	4.19	28.6	0.81	0.76	–0.065	0.759	0.0369	0.3796
CH02g01	184–246	14	6.09	14.3	0.80	0.84	0.036	0.834	0.0113	0.3360
**Mean**	–	**14.6**	**5.94**	–	**0.78**	**0.81**	**0.036**	**0.811**	–	–
**Total**	–	278	112.92	–	–	–	–	–	**2.2** × **10**^-^**^38^**	**8.5** × **10**^-^**^10^**

*^†^RA: percentage of rare alleles; an allele is defined rare at a frequency < 0.01; ^‡^The total value of the column is the cumulative P_*(ID)*_ obtained as the product of the P_*(ID)*_ of individual *loci*. The same is for P_*(ID)sib*._*

Rare alleles were found in 17 *loci* and were more common as the number of alleles per *locus* increased. Rare alleles ranged from 12.5% (at *locus* CH04c07 rare alleles with a frequency less than 1% were 2 out of 16) to 57% (at *locus* CH-Vf1 they were 8 out of 14). No rare allele were found at the *loci* Hi22f12 and AU223657, where the range of allele size were the lowest, 16 and 13 bp, respectively (**Table 3**).

Except for CH-Vf1 and CH01c06, all other *loci* were not in Hardy-Weinberg equilibrium (*P* ranging from 0.05 to less than 0.001) and this was expected as the 175 individuals do not belong to a panmictic population. Mean observed heterozygosity (H_o_) was 0.78, ranging from 0.16 (*locus* Hi22f12) to 0.91 (CH01g12 and CH04c07) (**Table 3**). Mean expected heterozygosity (H_e_) was 0.81, denoting high variability and ranging from 0.65 (CH-Vf1) to 0.89 (Hi07h02 and Hi03a10). F coefficients ranged from - 0.154 (CH-Vf1) to + 0.784 (Hi22f12), but the latter was the only value significantly departing from the others, thus denoting high homozygosity.

The 19 *loci* showed PIC values ranging from 0.65 (CH-Vf1) to 0.89 (Hi07h02 and Hi03a10), which were all higher than 0.5 and therefore very informative (Botstein et al., 1980).

The probability of identity (P_ID_) of a *locus* is the probability that two individuals share the same genotype at that *locus*, while the power of discrimination (PD = 1-P_ID_) is the probability that two individuals have different genotypes at that *locus*. An overall mean value of PD = 0.98 (ranging from 0.924 to 0.997) indicates that the *loci* are polymorphic enough in discriminating individuals. By considering the profile of the 19 *loci* at the same time, the probability to find two identical individuals is indeed remote (*P*_ID_ = 2.2 × 10^-38^); therefore, two individuals with the same profile at 19 *loci* are expected to be clones of the same genotype.

### Genetic Structure and Identification of Unknown Accessions

The Ward’s clustering method, based on the Euclidean distance matrix, highlighted several individuals grouped at zero distance and, according also to the *P*_ID_ value based on the polymorphisms at 19 *loci*, they can be considered to be the same genotype (**Supplementary Figure S2**). **Table 4** lists 38 accessions that were found to be identical. Among these, accessions #049, #012, #058, #147, and #013 were identical to #087, #010, #059, #118, and #043, respectively; they confirmed the correctness of the procedure as they had the same names, even if some of them were provided by different collections. Moreover, five unknown accessions, namely #076, #173, #148, #151, and #093 were identical to known genotypes and could therefore be named Annurca, Fuji, Coccianese, Rosciola, and Reinette du Canada, respectively. Some other accessions found to be identical can be considered synonyms: #002 of 001_Cerina, #134 of 126_Limoncella, #015 of 011_Sona, #028 and #030 of 016_Gelata, #041 of 039_Ciocarina Bianca, #053 of 080_ Ducale, #112 of 146_Gelata, #035, and #036 of 034_Panaia. Lastly, there were 5 other pairs of identical accessions (#055 and #060; #095 and #160; #103 and #157; #099 and #140; #054 and #102), all of unknown origin. In order to proceed with the statistical analysis accessions #054, #060, #103, #140, and #160 were removed.

**Table 4:** Accessions with identical SSR profile at 19 *loci*, used to identify unknown accessions and synonyms^∗^.

Synonyms/Unknown	Reference accession	Cluster
049_Amerina	087_Amerina	1
012_Ruzza	010_Ruzza	2
058_Coccianese	059_Coccianese	3
147_Rosa in Pietra	118_Rosa in Pietra	3
013_San Giovanni	043_San Giovanni	5
148_Unknown	149_Coccianese	3
076_Unknown	136_Annurca	4
173_Unknown	171_Fuji	5
151_Unknown	082_Rosciola	5
*093_Unknown*	*131_Reinette du Canada*	5
112_Paradisa	146_Gelata	1
002_Zuccherina	001_Cerina	1
028_Cerina	016_Gelata	1
030_Gelata		
015_A Sonagli	011_Sona	3
041_Ciochera Rosa	039_Ciocarina Bianca	3
134_Limoncina	126_Limoncella	3
053_Rosa d’Amelia	080_Ducale	3
*035_Pagliaccia*	*034_Panaia*	4
*036_Casciola*		

*^∗^Please note that other five pair of Unknown accessions were identical and only one was maintained for further analysis (see text in Results). Accessions in italics are putative polyplois.*

**FIGURE 1:**
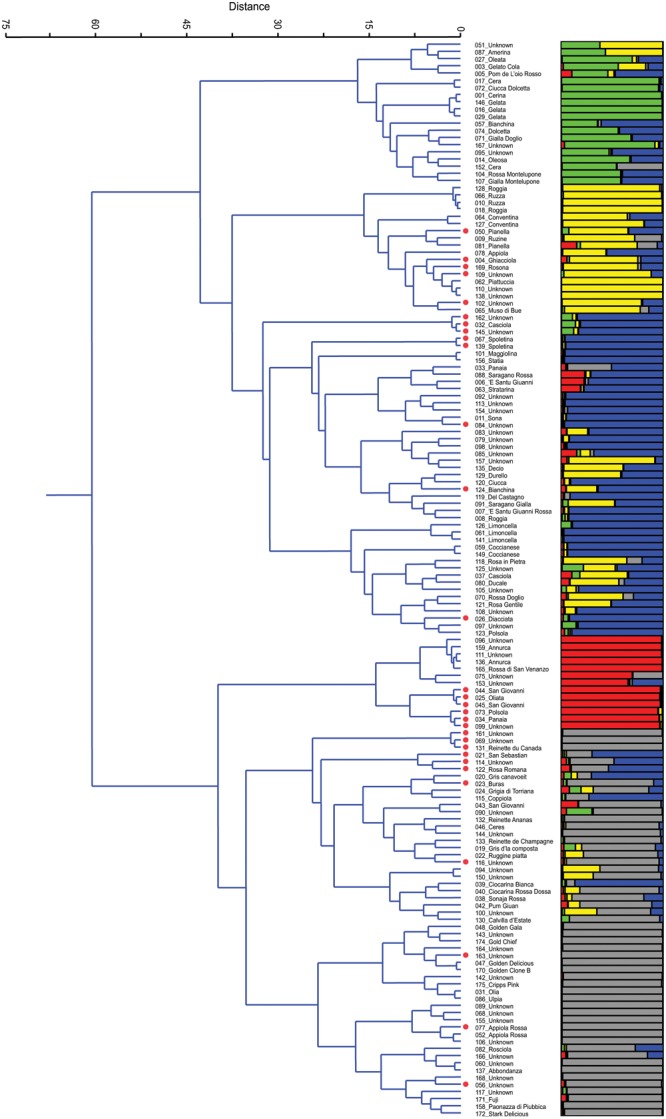
Ward’s clustering dendrogram of the 150 accessions of apple (on the left) and assignment by STRUCTURE at *K* = 5 (on the right). Polyploids are red labelled beside the accession name.

After the removal of 25 duplicates, the 150 accessions were reanalyzed by Ward’s clustering and analyzed by STRUCTURE. The results are reported in **Figure 1** and are aligned in order to compare and, eventually, validate accession membership and population structure. On Ward’s dendrogram and at a distance of 60 units we found two main groups (*P* < 0.001). The upper group includes all local varieties, while the other includes several commercial varieties that were used in this study as controls: Annurca, Reinettes, Abbondanza, Golden Delicious, Golden Gala, Fuji, Cripps Pink, and Stark Delicious. Moreover, at a distance of 35 the dendrogram showed essentially 5 clusters, in agreement with *K* = 5 of STRUCTURE (see later paragraph).

Cluster 1 is characterized by a group of local varieties which refer to ‘Gelata’ (#146, #016, #029), common in central Italy where it is known with different names: Cerina, Oleata, Cera (#001, #027, #017, and #152, respectively) (Gallesio, 1817/1839; Molon, 1901; Tamaro, 1929). In this cluster there are also two very similar accessions (#104 and #107) from Montelupone (Marche), differing from one another only for the skin color, red, and yellow, respectively.

Cluster 2 includes several ancient accessions from Umbria, such as Ruzza (#066 and #010) or Roggia (#128 and #018), which had already been described in the 16th century (Gallo, 1540). In this cluster there are two accessions named ‘Conventina’ (#064 and #127) from Gubbio (Umbria), which is the Italian name for ‘Monastery,’ and where this variety was grown since the Middle Ages, and it is known to be suitable for mountain areas (Tonini, 1930). Lastly, there is a subcluster of Piattuccia (#062) and Pianella (#050, #081) whose names are due to a flattened fruit shape.

Cluster 3, with 45 accessions, includes the subgroups of Spoletina (#067, #139), of Limoncella (#126, #061, and #141), of several polyploids (red labeled beside the name), the local varieties Sona (#011), Coccianese (#059) and several unknown accessions. Limoncella is an ancient variety common in the South of Italy whose name means ‘small lemon’; Sona is common in Valnerina (Umbria) and its name derives from the rattling sound of the seeds inside the carpel detaching at maturity.

Although apparently uniform, in Cluster 4 it is possible to detect two subgroups, one including all diploids accessions that refer to Annurca, an ancient cultivar of Campania whose origins were already described by Pliny the Elder (Pasquale, 1876), and the other including all polyploids and refers to Panaia (#034) and Polsola (#073), a synonym of Panaia, a local variety from Tuscany that spread in Umbria around 1700 and later in Abruzzo (Gallesio, 1817/1839). In the Annurca subgroup there are two unknown accessions, #096 and #111, tightly joined together (*P* < 0.06), both collected in Umbria.

Cluster 5 is the most numerous. It includes 54 accessions and it is possible to distinguish the group of Reinettes, the group of Golden, that of Appiola, that of Abbondanza and that of Fuji, Cripps Pink, and Stark Delicious. The Reinette accessions (#131, #132, and #133) and those known as ‘Mele Grigie’ (‘gray apples,’ from #019 to #024), provided by the Department of Agriculture of Torino, are very common in the North-West of Italy and are characterized by an acidic pulp and by a shrunken skin at maturity.

The plot of the average log-likelihood values for *K*s ranging from 1 to 21 and the distribution of Δ*K*-values (Evanno et al., 2005) according to *K*-values are shown in the **Supplementary Figure S3**. Two peaks were found, the first corresponding to *K =* 2 and the second to *K =* 5; the hierarchical genetic structure was investigated at *K =* 5. A threshold value of *P*_qI_ ≥ 0.80 was used to assign individuals to the groups (**Figure 1**). With this threshold as many as 58 of 150 individuals were classified as admixture. At *K* = 5 STRUCTURE was able to define 5 groups, identified by the colors green, yellow, blue, red, and gray, respectively, which are represented side by side with those detected by Ward’s clustering (**Figure 1**). By comparing the two grouping methods, and apart from the admixtures, it can be stated that there was a similar trend and correspondence. In Group 1 (green) only 7 out of 20 accessions were not classified as admixtures, and they all belong to Gelata and Cera (these are normally considered synonyms). In Group 2 (yellow) only 7 out of 18 accessions were classified at *P*_(qI)_≥ 0.80; of these, some belong to the group of Ruzza (synonym of Roggia, #128, #066, #010, #018, Dalla Ragione and Dalla Ragione, 2011) and the rest to Piattuccia (#062, #110, #138), confirming their cluster closeness. In Group 3 (blue), the cluster of Spoletina, Sona and Limoncella, 28 out of 45 were correctly classified (*P*_(qI)_ ≥ 0.80). In Group 4 (red) 11 out of 13 accessions were correctly classified: the Annurca’s (all diploids) and a set of triploids, all belonging to #034_Panaia. Group 5 (gray) includes several commercial varieties (Reinettes, Golden Delicious, Golden Gala, Abbondanza, Fuji, Cripps Pink, and Stark Delicious) and here as many as 35 out of 54 accessions were accurately classified. Overall, by comparing the two methods, only two misclassified accessions emerged: the unknown #157 found in Ward’s Cluster 3 but ascribed by STRUCTURE in Group 2 (yellow), and 039_Ciocarina Bianca in Cluster 5 attributed by STRUCTURE in Group 3 (blue).

Having established that 92 accessions showed a genetic structure, the five groups were compared in terms of number of alleles, expected and observed heterozygosity (**Table 5**) and F-Statistics (**Table 6**). However, the mean number of alleles across the 19 *loci* and for all 92 accessions, as revealed by STRUCTURE, was 13.1, very similar to the population based on 150 accessions (14.6); likewise were the values of H_e_ (0.82 for both) and H_o_ (0.79 *vs*. 0.78, respectively). As expected, the mean number of alleles per *locus* in Groups 1, 2, and 4 was consistently lower than those for Groups 3 and 5, since heavily dependent on the number of accession forming the groups. Interestingly, in the former, less numerous groups, there was at least one *locus* with a fixed allele (**Table 5**), hence the indexes of diversity (H_e_) were lower than those of Group 3 and 5. As expected, the overall F_IS_ of the 5 groups was slightly negative (outbreeding), consistent for the majority of the *loci* (14), except for CH05e03 (0.1045, *P* < 0.05) and especially for Hi22f12 (0.7914, *P* < 0.001), highly homozygous compared with the expected values. The overall *loci F*_ST_-value (0.1470, *P* < 0.001) is to be considered rather high, meaning that the 92 accessions were well structured and close to a value (0.15), generally considered to indicate a threshold limit between a moderate and a great differentiation (Wright, 1978). However, upon closer inspection of the *F*_ST_-values at each *locus* it is possible to note that, apart from those *loci* whose values are close to the mean, some exceeded it by at least 3 times the standard error; these were CH05c06, Hi22f12, and AU223657 with 0.1995, 0.2162, and 0.3074, respectively, all significant at *P* < 0.001. Some other *loci* showed values more than 3 times lower than the mean; they were CH04c07, CH01h01, CH-Vf1, and CH01f03b, all significant at *P* < 0.001. In particular *locus* CH01h02 with a *F*_ST_-value of 0.0403 (*P* < 0.05) denoted a little differentiation. All the results reported above were confirmed by AMOVA, where the variation within groups was 75.9% and among was 24.1%, values different from those reported in literature (Gasi et al., 2010; Urrestarazu et al., 2012, 2016; Pereira-Lorenzo et al., 2017).

**Table 5:** Number of alleles (Na), observed (H_o_), and expected (H_e_) eterozygosity for the five groups revealed by STRUCTURE.

	ALL (*n* = 92)			Group1 (*n* = 7)			Group2 (*n* = 9)			Group3 (*n* = 29)			Group4 (*n* = 11)			Group5 (*n* = 36)		
Locus	Na	H_e_	H_o_	Na	H_e_	H_o_	Na	H_e_	H_o_	Na	H_e_	H_o_	Na	H_e_	H_o_	Na	H_e_	H_o_
CH05c06	13	0.81	0.83	3	0.60	1.00	1^a^	0.00	0.00	10	0.77	0.90	6	0.73	1.00	9	0.79	0.89
CH-Vf1	10	0.64	0.74	2	0.49	0.71	2	0.11	0.11	7	0.71	0.90	3	0.65	1.00	7	0.63	0.69
Hi07h02	15	0.89	0.87	3	0.38	0.43	3	0.66	1.00	14	0.92	0.86	5	0.77	1.00	11	0.82	0.89
CH03d12	22	0.87	0.78	2	0.14	0.14	5	0.71	1.00	16	0.89	0.93	7	0.80	0.75	10	0.74	0.75
CH05e03	22	0.90	0.70	2	0.54	1.00	6	0.64	0.67	14	0.89	0.66	2	0.42	0.00	13	0.90	0.89
Hi04e04	14	0.88	0.85	2	0.44	0.57	3	0.45	0.56	9	0.83	0.97	5	0.70	1.00	9	0.86	0.83
CH02b03b	13	0.86	0.90	2	0.53	0.86	4	0.69	1.00	8	0.76	0.86	6	0.78	1.00	10	0.83	0.89
CH01f03b	11	0.79	0.89	4	0.71	1.00	4	0.69	1.00	8	0.81	0.90	3	0.50	0.55	8	0.81	0.94
CH02c09	12	0.85	0.85	4	0.67	1.00	1^b^	0.00	0.00	8	0.81	0.86	6	0.83	1.00	8	0.82	0.97
Hi22f12	5	0.76	0.13	3	0.54	0.14	3	0.57	0.11	5	0.75	0.18	1^c^	0.00	0.00	4	0.73	0.14
CH01g12	17	0.88	0.91	3	0.67	1.00	5	0.60	0.78	12	0.84	0.90	5	0.70	1.00	11	0.84	0.92
AU223657	6	0.78	0.67	2	0.26	0.29	3	0.66	1.00	5	0.78	0.79	3	0.63	1.00	4	0.47	0.47
CH01h02	9	0.71	0.75	3	0.66	0.86	5	0.71	1.00	8	0.74	0.72	3	0.45	0.55	5	0.72	0.75
CH01h01	11	0.85	0.88	2	0.36	0.43	4	0.71	1.00	11	0.87	0.90	5	0.78	1.00	7	0.81	0.89
CH04a12	14	0.80	0.80	3	0.66	0.86	3	0.57	0.89	10	0.81	0.76	4	0.46	0.55	8	0.80	0.89
Hi03a10	15	0.88	0.75	3	0.63	0.71	3	0.69	1.00	13	0.90	0.66	4	0.61	0.73	9	0.76	0.78
CH04c07	16	0.89	0.92	3	0.69	1.00	4	0.71	1.00	12	0.83	0.90	7	0.87	1.00	9	0.85	0.89
CH01c06	11	0.76	0.83	1^d^	0.00	0.00	3	0.66	1.00	9	0.75	0.93	3	0.50	0.55	8	0.83	0.94
CH02g01	13	0.82	0.77	3	0.69	1.00	3	0.63	0.89	9	0.80	0.86	4	0.71	0.55	8	0.74	0.69
Mean	13.1	0.82	0.78	2.6	0.51	0.68	3.4	0.55	0.74	9.9	0.81	0.81	4.3	0.63	0.75	8.3	0.78	0.80

*^*a*^*locus* with a fixed allele 122; ^*b*^*locus* with a fixed allele 248; ^*c*^*locus* with a fixed allele 218; ^*d*^*locus* with a fixed allele 163.*

**Table 6:** F statistics and significance for each *locus* and total of the 5 groups as obtained by STRUCTURE (SPAGeDi).

Locus	*F*_IS_^‡^	*F*_ST_
CH05c06	–0.2264 ^∗∗∗^	0.1995^∗∗∗^
CH-Vf1	–0.2398 ^∗∗∗^	0.0894^∗∗∗^
Hi07h02	–0.1029 ^∗^	0.1346^∗∗∗^
CH03d12	–0.0687 n.s.	0.1659 ^∗∗∗^
CH05e03	0.1045 ^∗^	0.1546 ^∗∗∗^
Hi04e04	–0.1200 ^∗∗∗^	0.1635 ^∗∗∗^
CH02b03b	–0.1988 ^∗∗∗^	0.1423 ^∗∗∗^
CH01f03b	–0.1968 ^∗∗∗^	0.0670 ^∗∗∗^
CH02c09	–0.1791 ^∗∗∗^	0.1849 ^∗∗∗^
Hi22f12	0.7914 ^∗∗∗^	0.2162 ^∗∗∗^
CH01g12	–0.1736 ^∗∗∗^	0.1403 ^∗∗∗^
AU223657	–0.1676 ^∗∗∗^	0.3074 ^∗∗∗^
CH01h02	–0.1151 ^∗^	0.0403 ^∗^
CH01h01	–0.1458 ^∗∗∗^	0.1050 ^∗∗∗^
CH04a12	–0.1146 ^∗^	0.1166 ^∗∗∗^
Hi03a10	0.0006 n.s.	0.1547 ^∗∗∗^
CH04c07	–0.1363 ^∗∗^	0.1084 ^∗∗∗^
CH01c06	–0.2209 ^∗∗∗^	0.1360 ^∗∗∗^
CH02g01	–0.0646 n.s.	0.1324 ^∗∗∗^
Mean	–0.0866 ^∗∗∗^	0.1470^∗∗∗^
*SE*	0.0460	0.0127

*^‡^F_*IS*_ is the loss of heterozygosity due to inbreeding, F_*ST*_ is the loss of heterozygosity due to genetic drift; ^∗, ∗∗, ∗∗∗^*F*-values significant at *P* < 0.05, 0.01, and 0.001, respectively; n.s. not significant.*

### Parentage Analysis

The parentage analysis was used (i) to investigate the origins of the 49 unknown accessions after the removal of 10 found identicals to well-known genotypes and (ii) to look for concordance with the results from STRUCTURE and Ward’s clustering.

**Table 7** reports the 38 unknown accessions significantly related (LOD score > 5) to parents of known origin (LV and CV). As many as 27 of them showed full concordance with STRUCTURE and Ward’s clustering. The most likely parents of 10 unknown accessions, classified by STRUCTURE into the Admixture group, were classified also by Ward’s in the same group. Lastly, the 144_Unknown was included by STRUCTURE in group 5, whereas the most likely parent, 033_Panaia, was classified differently by the other two analytic procedures (Ward’s Cluster3 and Admixture in STRUCTURE).

**Table 7:** Parentage analysis of unknown accessions, their membership to the group of Stucture, the most likely parent of known origin (LOD score as otained by FaMoz) and its membership to Cluster and to STRUCTURE.

Unknown accessions	Classified by STRUCTURE into Group	Likely parents of known origin	LOD score	Ward’s Cluster Number	Classified by STRUCTURE into Group
#051	Adm	#001 Cerina	12.79	1	1
		#062 Piattuccia	11.56	2	2
#167	1	#016 Gelata	15.34	1	1
		#029 Gelata	14.65	1	1
		#146 Gelata	11.69	1	1
		#001 Cerina	11.00	1	1
#095	Adm	#001 Cerina	9.27	1	1
		#146 Gelata	5.00	1	1
#109	2	#062 Piattuccia	17.87	2	2
#110	2	#062 Piattuccia	22.50	2	2
		#004 Ghiacciola	16.29	2	2
#138	2	#062 Piattuccia	23.33	2	2
		#004 Ghiacciola	17.00	2	2
#102	Adm	#062 Piattuccia	17.91	2	2
#162	3	#032 Casciola	21.81	3	3
#145	3	#032 Casciola	23.40	3	3
#084	3	#011 Sona	22.84	3	3
#079	3	#135 Decio	14.69	3	3
#097	3	#123 Polsola	10.44	3	3
#096	4	#165 Rossa di San Venanzo	24.47	4	4
		#159 Annurca	23.52	4	4
		#136 Annurca	23.41	4	4
#111	4	#159 Annurca	24.27	4	4
		#136 Annurca	24.16	4	4
#075	Adm	#136 Annurca	16.78	4	4
#153	Adm	#159 Annurca	11.17	4	4
		#136 Annurca	11.05	4	4
#099	4	#073 Polsola	30.01	4	4
		#034 Panaia	27.96	4	4
#161	5	#131 Reinette du Canada	25.20	5	5
		#019 Gris d’la Composta	6.04	5	5
#069	5	#131 Reinette du Canada	25.88	5	5
		#019 Gris d’la Composta	6.72	5	5
#090	Adm	#043 San Giovanni PT	21.60	5	5
#144	5	#033 Panaia	8.72	3	Adm
#116	5	#131 Reinette du Canada	6.37	5	5
#094	Adm	#038 Sonaja Rossa	7.04	5	5
#150	Adm	#038 Sonaja Rossa	5.93	5	5
#100	Adm	#130 Calvilla d’Estate	14.04	5	5
#143	5	#047 Golden Delicious	19.31	5	5
		#170 Golden Clone B	18.74	5	5
		#174 Gold Chief	13.66	5	5
		#172 Stark Delicious	12.47	5	5
		#171 Fuji	7.99	5	5
		#048 Golden Gala	6.47	5	5
#164	5	#047 Golden Delicious	11.50	5	5
		#170 Golden Clone B	10.81	5	5
		#175 Cripps Pink	9.13	5	5
		#174 Gold Chief	5.58	5	5
#163	5	#170 Golden Clone B	16.89	5	5
		#047 Golden Delicious	13.56	5	5
		#174 Gold Chief	12.67	5	5
		#137 Abbondanza	6.82	5	5
#142	5	#086 Ulpia	15.32	5	5
		#031 Olia	13.71	5	5
#089	5	#052 AppiolaRossa	16.14	5	5
		#137 Abbondanza	13.24	5	5
		#077 Appiola Rossa	7.25	5	5
#068	5	#137 Abbondanza	19.26	5	5
		#052 AppiolaRossa	13.67	5	5
#155	5	#137 Abbondanza	16.43	5	5
		#052 AppiolaRossa	11.63	5	5
		#158 Paonazza di Piubbica	6.92	5	5
#106	5	#052 AppiolaRossa	30.40	5	5
		#077 Appiola Rossa	22.15	5	5
#166	Adm	#137 Abbondanza	19.49	5	5
#060	5	#137 Abbondanza	33.23	5	5
		#158 Paonazza di Piubbica	13.17	5	5
#168	5	#052 AppiolaRossa	15.94	5	5
		#172 Stark Delicious	15.77	5	5
		#077 Appiola Rossa	6.25	5	5
		#158 Paonazza di Piubbica	5.80	5	5
#056	5	#077 Appiola Rossa	8.41	5	5
		#172 Stark Delicious	8.00	5	5
		#052 AppiolaRossa	7.95	5	5
#117	5	#172 Stark Delicious	17.69	5	5
		#174 Gold Chief	6.22	5	5

In particular, 051_Unknown was classified by STRUCTURE as admixture, showing a probability of 0.38 to be assigned to Group1 and of 0.61 to Group2; likely parents were 001_Cerina and 062_Piattuccia, belonging to Cluster1/Group1 and to Cluster2/Group2, respectively. Since Ward’s clustering assigned #051 to Cluster 1, it can be stated that STRUCTURE was more efficient to infer its mixed genomic configuration.

### Correlation of SSR Alleles and Some Morphological Traits

Correlations among morphological traits were not significant, except over color *vs.* fruit rustiness (*r* = -0.3166, *P* < 0.001). Eleven out of 19 SSR *loci* revealed that 15 alleles out of 278 were significantly (*P* < 0.001) correlated with five morphological traits (**Table 8**). Four alleles showed a significant negative correlation with time of eating maturity, so that the presence of these alleles was related with early maturity. CH03d12_128 and Hi03a10_199 were positively correlated with fruit shape. Similarly, pulp color revealed two alleles with significant positive correlations with two SSR *loci* related to aroma compounds (Dunemann et al., 2009), indicating putative correlation between pulp color and aroma trait.

**Table 8:** Spearman correlation coefficients (*r*) and statistical significance (*P*-value) between SSRs *loci* and morphological traits.

Traits	SSR locus_allele	*r*	*P*-value
Time of eating maturity	CH05c06_106	–0.28146	0.0010
	CH02c09_234	–0.28809	0.0008
	CH01g12_146	–0.28636	0.0008
	AU223657_235	–0.28460	0.0009
Fruit shape	CH03d12_128	0.34733	0.0001
	Hi03a10_199	0.29298	0.0006
Ground color	CH01f03b_137	0.28016	0.0010
Over color	CH_Vf1_127	–0.36308	0.0001
	Hi04e04_222	–0.31808	0.0002
	CH02b03b_077	–0.30490	0.0004
	CH01g12_151	–0.31013	0.0003
	Hi03a10_253	–0.31440	0.0002
	CH01c06_159	–0.31436	0.0002
Pulp color	Hi04e04_247	0.38470	0.0001
	CH01g12_146	0.33177	0.0001

The over color was found negatively correlated with several alleles, with *r*-values ranging from - 0.3049 (for CH02b03b_077) to - 0.36308 (for CH_Vf1_127), meaning that their presence is by some means related to light colors (absence, yellow, and orange). Interestingly, the majority of the marker-alleles detected for the over color were related to QTLs for resistance to biotic stresses, aroma compounds, stiffness, and acidity, indicating a possible correlation among these traits (Kenis et al., 2008). Lastly, only one allele (CH01f03b_137) resulted positively correlated with ground color.

Moreover, three (CH01g12, Hi03a10, and Hi04e04) out of eleven *loci* identified in the correlation test were found in at least two traits, while CH01g12 was detected in maturity, over color and pulp color.

## Discussion

The rapid spread of modern, intensive agricultural techniques during the last century was also accompanied by a rapid spread of newly bred cultivars characterized by greater productivity and uniformity. Although this trend was more intensive in annual crops, it did not spare fruit orchards. The oversimplification of the agricultural systems in favorable areas caused parallel changes in the agricultural economy, farming assets, rural culture, and agricultural landscapes as well. From a biological point of view this determined a significant reduction of crop biodiversity and a progressive genetic erosion with the loss of many ancient, well adapted local varieties. For this reason, over the last 50 years, the need to develop effective strategies for the conservation and management of genetic resources has become a fundamental issue. At this purpose germplasm banks have been established all over the world, operating at international, national and local levels. Italy, in compliance with EU directives, has promoted several Regional germplasm collections, with the aim of maintaining and preserving the autochthonous diversity. In Italy fruit germplasm, and apple genetic resources in particular, are conserved by National and Regional institutions (such as CREA, University of Bologna, Malva Rinaldi School of Torino, and many others), several of whom kindly provided accessions that were used as controls in this study.

The area of Central Italy is characterized by hills, mountains, small valleys, a variety of soil types (Corti et al., 2013) and of exposure, generating many micro-environments. Rainfall amounts and distribution from the East to the West coasts, passing through the Apennines, is also different, as well as the temperatures due to altitude differences from sea level to 2000–3000 m a.s.l. (Longinelli and Selmo, 2003). Fruit in general, and apples in particular, were rarely grown in specialized and intensive cultivation over large areas. Therefore, coupling these conditions together can perhaps explain most of the reasons at the base of the rich diversity found in the apple germplasm of Central Italy, a picture difficult to find in other Italian regions.

Many of these local varieties are well adapted to specific agro-climatic conditions and often express some diversity with respect to the originals in terms of morphological and physiological traits, thus assuming different names. Often, the names were assigned on the base of phenotypic traits, strongly influenced by the environment and agricultural practice, thus increasing the existing confusion about local genetic resources and their correct denomination. This gives rise to the importance of characterizing the germplasm present in the national and regional germplasm banks, by identifying duplicates and redundant accessions, hence simplifying the management and reducing costs of living collections.

For this purpose, molecular markers and in particular SSR have been widely used in genetic diversity studies and clarified cases of synonymy and homonymy in core collections (Patzak et al., 2012; Urrestarazu et al., 2012, 2016; Liang et al., 2015; Yun et al., 2015; Lassois et al., 2016). Following the detection of null alleles, two of the initial 21 *loci* (Hi23g02 and Hi03g02) were discarded. The remaining 19 showed a high degree of polymorphism and discriminating power and allowed us to meet our objectives.

The pool of accession studied here showed a percentage of polyploids of 20%, a value intermediate between 8% reported by Urrestarazu et al. (2016) in screening a wide European collection, and those found in Spain: 34% by Pereira-Lorenzo et al. (2007), 29% by Ramos-Cabrer et al. (2007), and 24% by Urrestarazu et al. (2012).

In brief, our study showed that 25 accessions were duplicates, 9 had to be considered synonyms (**Table 4**) and 9 homonyms. Six accessions from the living collection of 3A-PTA Pantalla and 3A-PTA Casalina (Amerina c02_049/ c02_087, Coccianese c02_058/ c02_059, and Rosa in Pietra c02_147/c03_118) were duplicates of the same genotype, because the SSR profiles were identical throughout the 19 *loci*. Also San Giovanni (c02_013) and Ruzza (c02_012) turned out to be identical to the corresponding accessions provided by the Department of Agricultural Science, University of Bologna (c08) and the National Center of Fruit Tree Germplasm (c01), thus confirming the goodness of the analysis.

Among polyploids we found some accessions named Panaia or Polsola, and they are synonyms (Dalla Ragione and Dalla Ragione, 2011). This ancient variety, whose local name ‘Panaia’ derives from ‘bread basket,’ was very common in Central Italy and in the past two varieties were described by Gallesio (1817/1839): ‘Panaia massima’ and ‘Panaia a frutto piccolo.’ These denominations refer to the dimension of the fruit, and may explain the homonymy between #034 and #075 (polyploids with bigger fruit) vs. #033 (diploid with smaller fruit).

As far as homonymy is concerned, three accessions named ‘San Giovanni’ were also scattered among Clusters 4 and 5. This is a group of accessions whose local name is linked to the time of maturation (end of June), independently of other traits, one is diploid (#043, Cluster 5) while the other two were polyploids (#044, #045, Cluster 4). Other examples of homonymy include #008_Roggia (Cluster3), genetically different from the two Roggia listed in Cluster2; Appiola Rossa #052 (diploid) vs. Appiola Rossa #077 (polyploid) in Cluster 5, and Bianchina #057 (diploid, in Cluster1) vs. Bianchina #124 (polyploid, in Cluster 3).

Another significant result of our study was the genetic identification of several unknown accessions. Ten of them were excluded as duplicates of well-known accessions. By using different statistical approaches (Cluster, STRUCTURE and Parentage analysis) it was possible to assign 37 more accessions to known commercial or local varieties.

Lastly, it is worth mentioning that the 92 accessions found by the Bayesian analysis were well-structured at *K* = 5, where the *F*_ST_-value indicates a high differentiation among subpopulations, much higher than those reported in the literature (Pereira-Lorenzo et al., 2007; Gasi et al., 2010; Urrestarazu et al., 2012, 2016), indicating that the material from Central Italy is a genetic pool worthy of safeguarding and conservation. In particular we found that F_ST_ at some *loci* were very contrasting (see **Table 6**). The low *F*_ST_-values at *loci* CH04c07, CH01h01, CH-Vf1, CH01f03b and CH01h02 suggests that homogenizing selection across subpopulations reduces differentiation, whereas the high *F*_ST_-values at *loci* CH05c06, Hi22f12, and AU223657 suggest that selection for local adaptation is creating differentiation. In these cases we found that the allele 128 of CH-Vf1 is correlated with fruit over color and allele 137 of *locus* CH01f03b with fruit ground color, while allele 106 of CH05c06 and allele 235 of AU223657 are correlated with time of eating maturity (**Table 8**).

Unexpectedly, the observed heterozygosity at the *locus* Hi22f12 was significantly lower than the values at the other 18 *loci* (0.13 *vs*. 0.78), meaning that almost all individuals at this *locus* are homozygous. Actually, this is also the *locus* with the lowest number of alleles. The sequence of the Hi22f12 SSR *locus* was then used in the BLAST program analysis against the NCBI nr database, and we found that Hi22f12 is located inside the transcription factor IIE subunit 1-like gene (XM_008376494.2) of *Malus × domestica*, at the position 1187–1242. The expectation and the identity of the query against the reference gene was 2 × 10^-6^ and 85%, respectively. The stability needed by this gene explains this low polymorphism, perhaps confined to introns. It would be interesting to extend this investigation to other germplasm collections.

In the present study Hi22f12 and some other SSR *loci* resulted fixed; in particular, *loci* CH05C06 and CH02c09 showed a fixed allele in group 2 of STRUCTURE. Of these, CH05C06 is of particular interest, associated with a major QTL for fruit titratable acidity (TA) detected in the *Ma* region (Liebhard et al., 2003; Kenis et al., 2008). In the cross ‘Telamon × Braeburn,’ this QTL was mapped in the LG16, an interval between the markers CH05e04z and CH05c06 and explained 20–34% of the observed variance (Kenis et al., 2008). The *Ma* gene controls the level of malic acid in apples and many other fruits (Maliepaard et al., 1998; Liebhard et al., 2003; Xu et al., 2012). Indeed, acidity is one of the most important fruit traits and, in apples, it strongly affects quality and organoleptic characteristics. In fact, the balance between sugars and acids is the basis of the taste and flavor of fruit (Wu et al., 2007; Zhang et al., 2010) and is therefore of utmost importance in breeding programs (Visser and Verhaegh, 1978). Moreover, this *locus* seems to be associated with a second QTL (M2), coding for the aromatic compound β-damascenone (Dunemann et al., 2009), a potent aroma present in apples (Cunningham et al., 1986; Fuhrmann and Grosch, 2002) and other fruits (peaches and grapes) and beverages (coffee, beer, and wine), and is associated with descriptions such as “fruity-flowery,” and in particular “apple” and “baked apple” (Pineau et al., 2007). In our study the allele 120 at the *locus* CH05C06 is fixed in all accessions of Group 2 of STRUCTURE, the one hosting Conventina, Ruzza, Roggia, Rosona, and Piattuccia, all characterized by crispy, sugary, sour, and very aromatic pulp. In the same group 2 we found fixed also the allele 248 of the *locus* CH2c09 and this *locus* is linked to a QTL for the aromatic compound allylanil (M1) associated with anise and licorice descriptors on the LG 15(Plotto and McDaniel, 2000; Dunemann et al., 2009). Two other allele in the present investigation, the 162 at *locus* CH01c06 and the 218 at *locus* Hi22f12, were also fixed in Group 1 and Group 4, respectively, but no information was found in the *Malus* database.

## Conclusion

In conclusion, this paper highlights the presence of considerable genetic variability among the apple accessions recovered in Central Italy and the information obtained can be used to better manage large living collections of a fruit tree of great nutritional interest such as the apple.

## Author Contributions

EA, LC, and FV conceived the study. EA and GM designed and coordinated the experiments. EA, GM, and LC chose and provided the germplasm. GM and NF performed the lab experiments. GM, LR, and NF conducted the data analysis and wrote the manuscript, while EA and FV critically reviewed it.

## Conflict of Interest Statement

The authors declare that the research was conducted in the absence of any commercial or financial relationships that could be construed as a potential conflict of interest.
