# Supplementary figures and images for: Genetic Characterization of the Apple Germplasm Collection in Central Italy: The Value of Local Varieties
Source: Front Plant Sci. 2018 Oct 10;9:1460. doi: 10.3389/fpls.2018.01460 (PMC6191466; doi:10.3389/fpls.2018.01460)

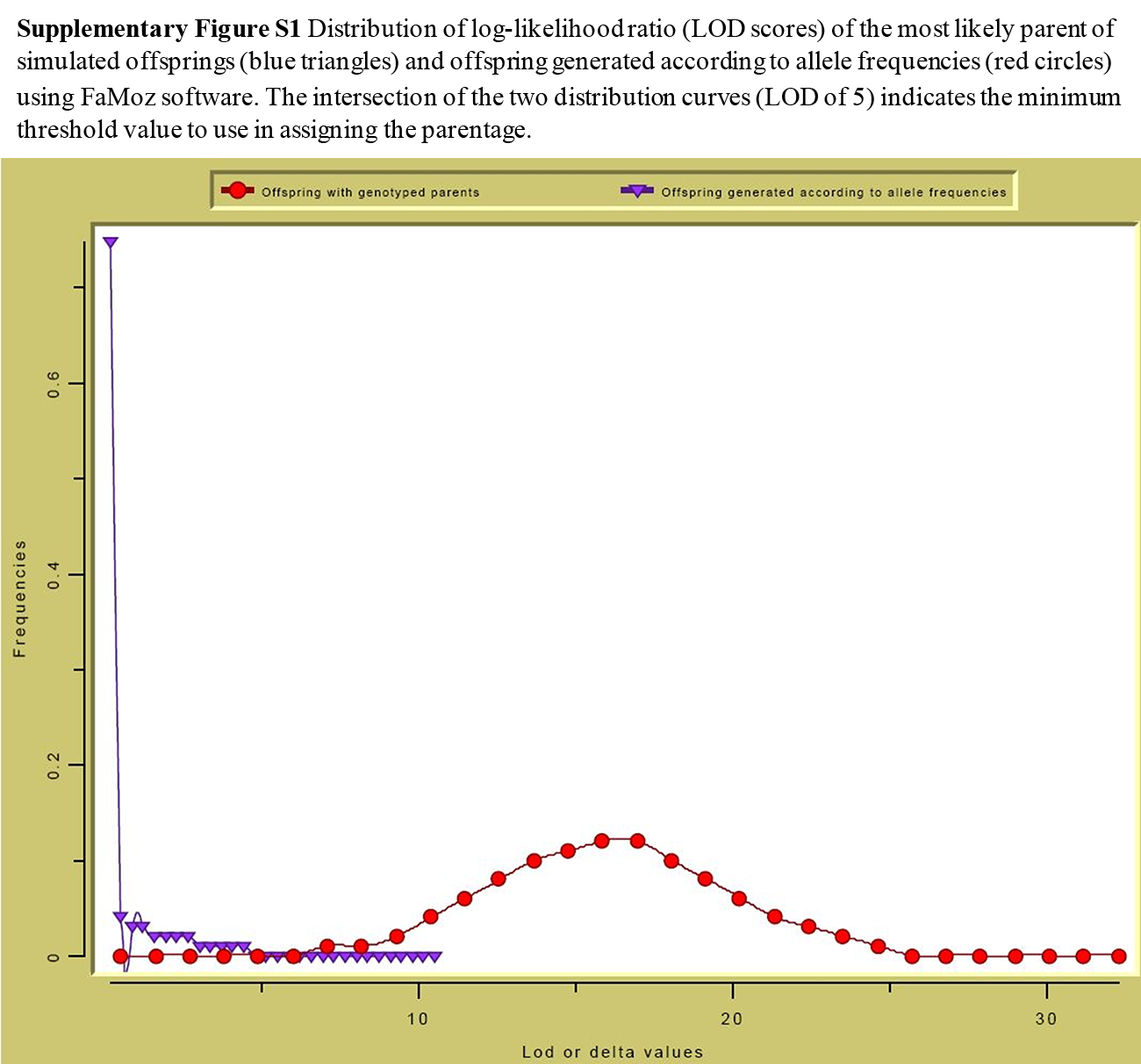

Supplement: Supplementary file 3 [file Image_1.TIF]

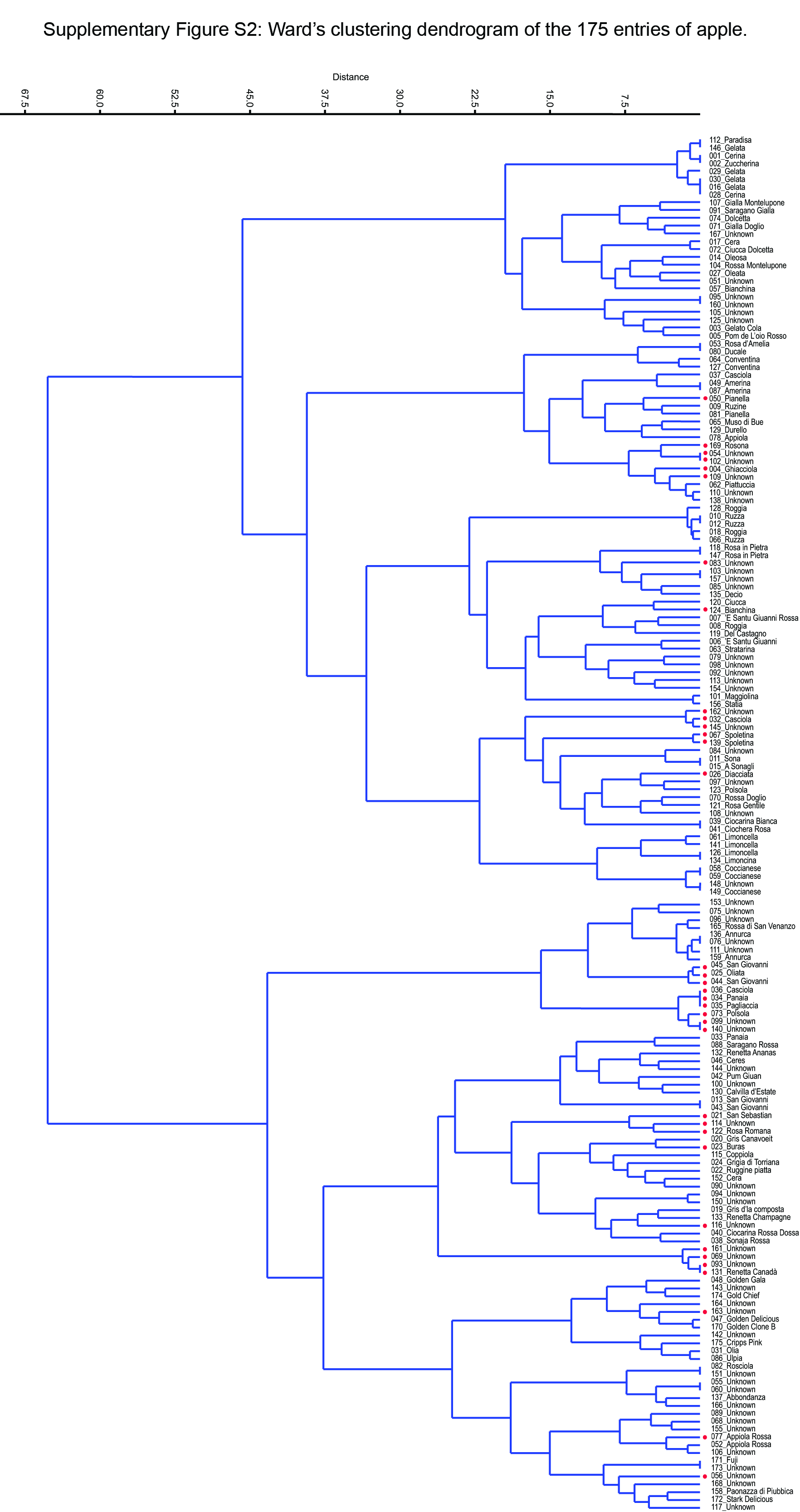

Supplement: Supplementary file 4 [file Image_2.TIF]

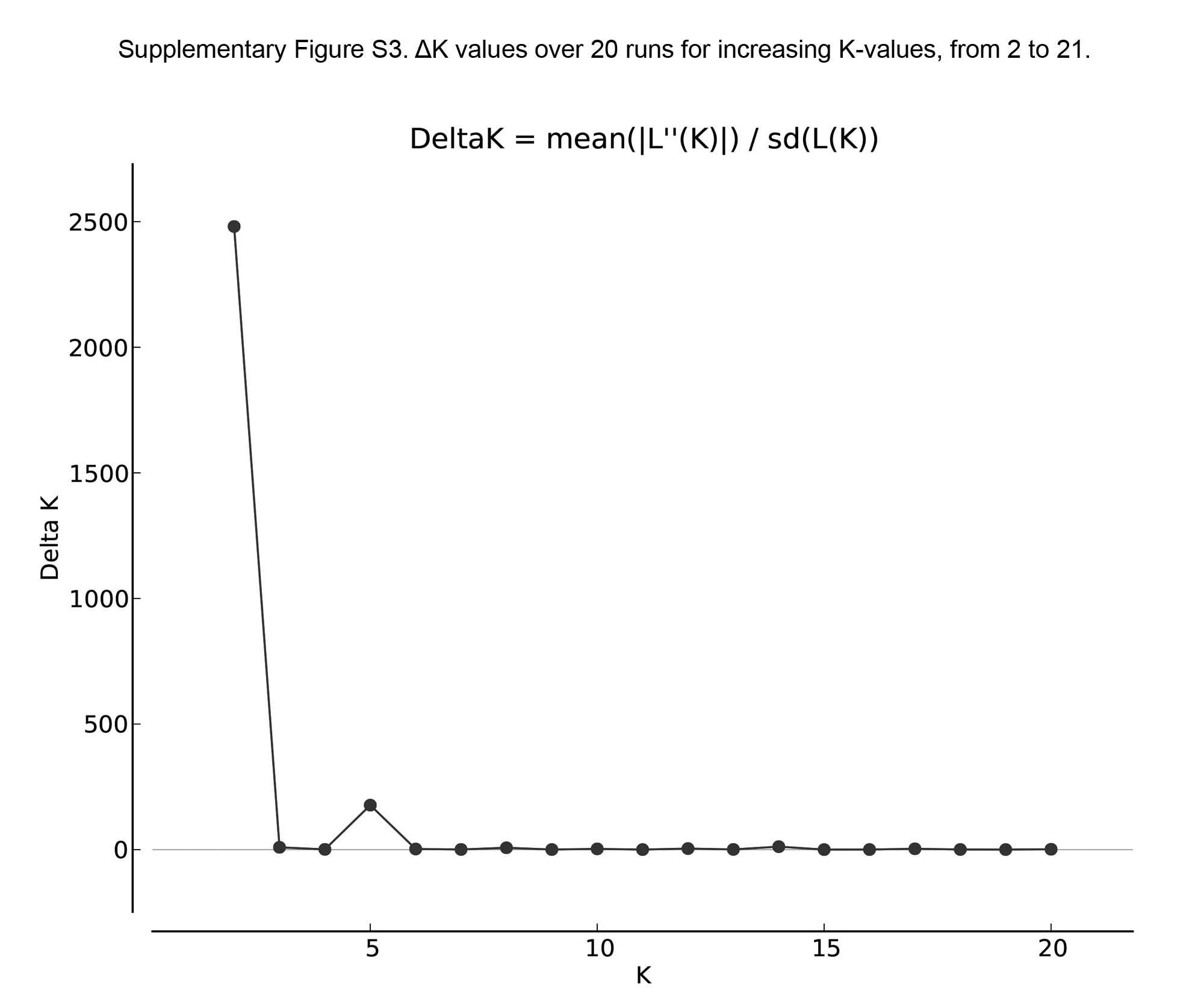

Supplement: Supplementary file 5 [file Image_3.TIF]
